# 607 Use of Autologous Skin Cell Suspension (ASCS) for Full-thickness Burn Injuries Reduces Autograft Procedures

**DOI:** 10.1093/jbcr/irac012.235

**Published:** 2022-03-23

**Authors:** James H Holmes, Kevin N Foster, Booker King, Rajiv Sood, Jeffrey E Carter, William L Hickerson

**Affiliations:** Atrium Health Wake Forest Baptist, Winston-Salem, North Carolina; The Arizona Burn Center Valleywise Health, Phoenix, Arizona; UNC Jaycee Burn Center, Chapel Hill, North Carolina; JMS/BRCA, Augusta, Georgia; University Medical Center- New Orleans, New Orleans, Louisiana; Spectral MD, Avita Medical, AccessPro Med, Memphis, Tennessee

## Abstract

**Introduction:**

Pediatric burn patients and patients with large TBSA injuries are vulnerable to morbidity and mortality, requiring multiple autograft (AG) procedures to obtain definitive closure due to limited donor site availability. The primary objective of this study was to understand the impact of ASCS, as an AG-sparing technology, on the number of AG procedures required to achieve definitive closure of full-thickness (FT) acute thermal burns.

**Methods:**

Retrospective analyses of real-world data were conducted, evaluating clinical outcomes of ASCS-treated patients in prospective, uncontrolled observational studies compared to control data from patients in the National Burn Repository version 8.0 (NBR) who had conventional AG (SOC). The pediatric population consisted of patients < 18 years of age, inclusive of any size TBSA, and the adult cohort included patients ≥ 18 years of age with >50% TBSA injury. Propensity score stratification was used to reduce bias attributable to potential differences in age, sex, %TBSA, and Baux scores between nonrandomized cohorts. Clinical outcomes evaluated included number of AG treatments, length of stay (LOS), healing, and mortality. Additional adverse events were evaluated and compared to historical SOC data sets.

**Results:**

The median number of AG procedures for pediatric patients treated with ASCS and control NBR cohorts was 1.0 (1.0-5.0) and 2.0 (1.0-20.0), respectively. For adult patients, the ASCS-treated and NBR cohorts had medians of 2.0 (1.0-6.0) and 5.0 (1.0-32.0) treatments, respectively. Overall, ASCS lead to approximately 60% fewer mean AG procedures for both populations. By week 8, re-epithelialization was observed in 91.8% and 90.6% of wounds in the pediatric and adult ASCS-treated cohorts. Median LOS for both cohorts were not different between the treatment groups. No significant differences were observed for mortality between cohorts and no adverse events attributed to ASCS were reported.

**Conclusions:**

ASCS treatment reduced the number of AG procedures needed to achieve closure, benefiting patients and offering burn centers reduced complexity and cost in the patient care pathway. While LOS was not significantly reduced in this study as seen in other reports, this finding may be confounded due to potential differences in the patient cohorts relative comorbidities and polytrauma, as well as variability in clinical site inpatient/outpatient management strategies for OT/PT.

